# Enhanced response of thermospheric cooling emission to negative pressure pulse

**DOI:** 10.1038/s41598-024-60471-2

**Published:** 2024-04-26

**Authors:** Tikemani Bag, Yasunobu Ogawa

**Affiliations:** National Institute of Polar Research, 10-3, Midori-cho, Tachikawa, Tokyo 190-8518 Japan

**Keywords:** Magnetospheric physics, Magnetospheric physics

## Abstract

Nitric oxide (NO) emission via 5.3 µm wavelength plays dominant role in regulating the thermospheric temperature due to thermostat nature. The response of NO 5.3 mm emission to the negative pressure impulse during November 06–09, 2010 is studied by using Sounding of Atmosphere by Broadband Emission Radiometry (SABER) observations onboard the Thermosphere Ionosphere Mesosphere Energetics and Dynamics (TIMED) satellite and model simulations. The TIMED/SABER satellite observations demonstrate a significant enhancement in the high latitude region. The Open Geospace General Circulation Model (OpenGGCM), Weimer model simulations and Active Magnetosphere and Planetary Electrodynamics Response Experiment measurements exhibit intensification and equatorward expansion of the field-aligned-currents (FACs) post-negative pressure impulse period due to the expansion of the dayside magnetosphere. The enhanced FACs drive precipitation of low energy particle flux and Joule heating rate affecting whole magnetosphere–ionosphere–thermosphere system. Our study based on electric fields and conductivity derived from the EISCAT Troms${\o }$ radar and TIEGCM simulation suggests that the enhanced Joule heating rate and the particle precipitations prompt the increase in NO cooling emission.

## Introduction

The Earth’s magnetosphere acts as a protective layer against the hazardous solar radiation and particles. However, the solar wind-magnetosphere interactions can lead to phenomena that affect the whole magnetosphere–ionosphere–thermosphere (MIT) system enveloping the near-Earth space environment. Since Earth’s magnetosphere is immersed in the solar wind, the solar wind dynamic pressure directly controls the size and configuration of Earth’s magnetosphere. The interplanetary (IP) shock is one of the common phenomena in solar wind that can result in the sudden change in the solar wind parameters. A shock is classified as fast or slow depending on the relative speed between shock and ambient solar wind; if it is greater than zero it is named as fast shock and slow shock otherwise. The fast shocks moving away from the sun are the fast forward shock (FFS), whereas, those moving towards the sun are known as fast reverse shock (FRS). Among all, FFSs are more frequent and relatively more geoeffective than FRSs, thereby, strongly impact MIT system. The shocks result in the discontinuity in solar wind parameter. For example, all solar wind parameters such as plasma density, magnetic field, temperature, and speed exhibit positive jumps in case of FFS. Whereas, there is decrease in the solar wind density and pressure, and increase in the magnetic field and solar wind speed during slow reverse shock (see Oliveira^1^ for a detailed review).

The abrupt increase (decrease) in the dynamic pressure results in the compression (expansion) of the magnetosphere. The study by Araki^2^ anticipated that the impacts of pressure impulse would be quickly countered by Field-Aligned-Current (FAC) and an opposing set of vortices. However, recently Gillies et al.^3^ suggested that the effect of pressure impulse continues till the compression ends. The pressure impulse (both positive and negative) strongly affects the electrodynamics of the magnetosphere and prompts many changes including current system, particle acceleration, convection and transport processes^4–7,]^and references therein. For example, Araki^2^ observed a transient enhancement in the H-component of low latitude geomagnetic field during positive pressure impulse. Similarly, a strong intensification of auroral luminosity in the local noon and subsequent propagation into the nightside including widening of auroral oval and reduction of polar cap has been reported during both positive and negative pressure impulse events^8–12,]^ and references therein. Further, generation of the high speed ($$\sim$$ several km/s) travelling convection vortices in high latitude ionosphere that propagates tailward away from local noon has been reported earlier^13–17^. In addition, the positive impulse has been observed to impact the ionospheric electron density, convection pattern, FAC and geomagnetically induced current^3,11, 18–21^. Although there is a general agreement that rapid dynamic pressure change happening over 10s of minutes is considered as sudden impulse (SI), there is some ambiguity in the literature about it’s characterization. Zuo et al.^22^ defines abrupt change of the dynamic pressure exceeding a given threshold value dp$$_0$$ = 1 nPa as SI. Other studies apply fractional change $$\frac{\delta P_{dyn}}{\left\langle P \right\rangle }>$$1 as threshold for SI^23,24^. Coco et al.^19^ defines SI as a change happening in 10 min or less. Zuo et al.^22^ considered the time duration of about 5 min (dt$$_0$$ = 5 minutes). Whereas, Vidal-Luengo et al.^24^ used less than 3 minutes as the criteria for SI. Hori et al.^11^ and Nishimura et al.^25^ represented the gradient of SYM-H as parameter for SI without limiting the time duration. Similarly, Gillies et al.^3^ accustomed the gradient of field. There is a strong disagreement about the definition of SI and is still under debate. We use both dynamics pressure and SYM-H index to define negative SI with the condition that $$\frac{dP_{dyn}}{dt}<$$ -0.25 nPa/minute and $$\frac{dSYMH}{dt}<$$-1 nT/ minute at least for 30 minutes in the present study. We would like to mention here that the event considered in the present study is of considerably longer duration than those reported by previous authors. Although previous studies revealed that it has the mirror-image relationship with positive pressure impulse (SI$$^+$$,^26–29^), the impacts of negative pressure impulse (SI$$^-$$) on MI system is not extensively studied as compared to its positive counterpart^30^. There is real dearth of compressive study on the impacts of negative dynamic pressure impulse on MIT system.

The thermospheric radiative emission by Nitric Oxide via 5.3 $\upmu$m wavelength plays a significant role in regulating thermospheric temperature during disturbed time^31^ and is well known as natural thermostat^32^. It results due to the vibration-rotation transition of nitric oxide molecule,

1 $$\begin{aligned} NO(\nu =1) \rightarrow NO(\nu =0)+5.3~ \upmu {\rm m }\end{aligned}$$

Above 100 km, the strong temperature dependent inelastic collision between atomic oxygen and nitric oxide density is the main production source for NO emission as follows,

2 $$\begin{aligned} NO(\nu =0)+O \rightarrow NO(\nu =1)+O \end{aligned}$$

Consequently, the NO emission depends linearly on the abundance of nitric oxide and atomic oxygen, and non-linearly on thermospheric temperature^32^.

In high latitude, the auroral particle flux creates nitric oxide density. The electrons of 1–10 keV and ions of energy 10–20 keV dissociate N$_2$ to produce N$(^2$D). The auroral electrons of 0.3–0.9 keV results in the formation of N($^4$S); where “D” and “S” represent the electronic states. They subsequently produce nitric oxide density due to the temperature dependent reaction with molecular oxygen^33,34^ as follows,

3 $$\begin{aligned} N(^4S,^2D)+O_2 \rightarrow NO+O \end{aligned}$$

In addition, the inelastic collision of molecular oxygen density with atomic nitrogen (N$^2$D) also produces NO in low latitude region^33–37^. During space weather events, the thermospheric radiative emission shows strong correspondence with the external energy deposition into the magnetosphere. Sounding of Atmosphere by Broadband Emission radiometry (SABER) observations onboard NASA’s Thermosphere Ionosphere Mesosphere Energetics and Dynamics (TIMED) satellite revealed that nitric oxide at 5.3 $\upmu$m accounted for about 50$\%$ of energy input during April 2002 geomagnetic storm^38^. Similarly, by using the Thermosphere-Ionosphere-Electrodynamics General Circulation Model (TIEGCM) simulation and TIMED/SABER observations, Lu et al.^39^ concluded that nitric oxide emission accounts for majority of Joule heating energy during a typical storm period. During non-storm period, the radiative cooling balances the EUV/UV heating and chemical heating. It also strongly dictates the thermospheric temperature and density^40^. There are several studies on thermospheric cooling emission during disturbed periods^41,42,]^and references therein. However, the response of NO emission to the dynamic pressure impulse is not yet explored. We, for the first-time, study MIT coupling during the negative pressure impulse, that developed under the non-storm period of November 06–09, 2010, by combining numerical model simulations and measurements.

## Method and data

The SABER observations of NO cooling emission onboard the TIMED satellite along with numerical models and measurements for field-aligned-currents (FACs) and Joule heating rates are utilized to investigate the response of NO emission to negative pressure impulse during November 06–09, 2010.

SABER is a limb sounder onboard the TIMED satellite. It scans Earth’s atmosphere from about 400 km to surface and back to measure radiance (W m$^{-2}$ sr$^{-1}$) in ten distinct spectral channels in the range of 1.27–16 $\upmu$m. SABER has asymmetrical coverage of hemisphere from about 53$^\circ$ latitude in one hemisphere to about 83$^\circ$ latitude in another, due to anti-sunward view, that changes in every 60–65 days. During these 60-65 days, SABER completes 24-h local time. SABER observes from the polar region in one hemisphere to high latitude in opposite hemisphere during one orbit. Over a day, it has about 15 orbits covering 15 longitude bands. The altitudinal profiles of NO 5.3 $\upmu$m volume emission rate (W m$^{-3}$) is calculated by applying an Abel inversion technique to the observed limb radiance (see Mlynczak et al.^32^ for details). The vertical profile is again integrated in the altitude of 100 to 250 km to get the cooling flux (W m$^{-2}$). The accuracy of the estimated NO cooling rate is better than 15$\%$^43^. In the present study we used the processed data version 2.0 obtained from SABER database via (saber.gats-inc.com/data.php).

The electron density, ion/electron temperature, Pederson conductivity and electric fields are obtained from the EISCAT Ultra High Frequency radar over Troms${\o }$ (geographic coordinates: 69.59$^\circ$ N, 19.22$^\circ$ E), Norway, in common program mode. The basic range and the temporal resolution of the measured ionospheric parameters above 70 km are controlled by the chosen pulse codes. The electric field is obtained from the tristatic measurements in F-region with the IGRF-magnetic field model applied to the $\vec {E}\times \vec {B}$ drift. The electric field has about 6 min time resolution in the monostatic mode. The dominant neutral parameter from the NRLMSISE (Naval Research Laboratory Mass Spectrometer and Incoherent Scatter Radar)-00 model^44^ and ions profiles along with the measured electron density are used to calculate the altitude profile of the Pederson conductivity in 1 min temporal and 1 km spatial resolutions^45,46^. The altitude-integrated Joule heating rate is calculated by using only the electric field part,

4 $$\begin{aligned} JH=\sigma _p. {\vec {E}}^2 \end{aligned}$$

where $\sigma _p$ is the Pederson conductivity in the altitude of 70-330 km and $\vec {E}$ is the electric field measured in the coordinate system fixed to the Earth.

The Field-Aligned-Currents (FACs) are obtained the Open Geospace General Circulation model (OpenGGCM,^55^), Weimer models^47^ and Active Magnetosphere and Planetary Electrodynamics Response Experiment (AMPERE^48–53^). Whereas, the Joule heating rates are estimated from the OpenGGCM and Weimer models, Thermosphere–Ionosphere-Electrodynamics General Circulation Model (TIEGCM) and EISCAT measurements. The OpenGGCM, Weimer and TIEGCM models were run on NASA’s Community Coordinated Modeling Center (https://ccmc.gsfc.nasa.gov/). The AMPERE dataset provides a continuous and global scale measurement of magnetic field perturbations due to FAC and upward and downward FACs on the magnetic latitude-magnetic local time grid by using Iridium satellite constellation. The FACs have 10 min integration time and 2 min time resolution^54^ ,i.e., the data are sampled in a new latitude $\times$ LT bin in every 2 min, but all bins are resampled once every 10 min.

The OpenGGCM is global magnetohydrodynamic (MHD) model that covers the magnetosphere from 20R$_E$ ($R_E$ = Earth’s radius) on the sunward direction to several hundreds R$_E$ on the anti-sunward side and about 48R$_E$ on y/z direction^55^. It solves the global MHD equations for Earth’s magnetosphere outside 3R$_E$ which is then coupled, from 3R$_E$ to Earth to magnetosphere, with the Coupled Thermosphere Ionosphere Model. The inner magnetosphere is coupled to the ionosphere via field-aligned-current. The ionospheric potentials are solved on a sphere which are then mapped into 58$^\circ$ to 90$^\circ$ magnetic latitude from 3R$_E$. Real time solar wind data from solar monitoring satellite or generic solar wind conditions can be used as input for this model. The OpenGGCM simulation is run with the input from the WIND satellite projected to 33 R$_E$ in GSM coordinates and Ring Current Model. The output is in GSE coordinate system. The OpenGGCM has been successfully used to simulate the impact of fast forward shocks on the magnetosphere^56,57^. In the present study, for the first time, the OpenGGCM model is used to investigate the impact of a slow reverse shock on the Earth’s magnetosphere.

The Weimer 2005^47^ is an empirical model that calculates high latitude electric potentials, FACs and Joule heating rate as function of solar wind parameters. It uses the scalar magnetic Euler potentials/electric potential for the calculation of FAC. Whereas, the Joule heating rate is derived from the magnetic FAC and electric potentials (see, Weimer^47^ for detailed discussion).

The TIEGCM is a time-dependent, three-dimensional model that solves the coupled non-linear, thermodynamic and hydrodynamic continuity equations for the neutrals, ions and wind, including the ion energy and momentum self-consistently^58^. The high-latitude precipitation and convection patterns, which represent the geomagnetic forcing, are obtained from Weimer model in the present study. The TIEGCM uses the formulation of Kockarts^31^ to calculate thermospheric cooling emission. The Pederson conductivity is calculated following Schunk and Nagy^59^ which uses collisional and gyrofrequency of dominant atmospheric species such as O, O$_2$, N$_2$, O$^+$, O$_2^+$, NO$^+$ and Ne. The Joule heating rate is estimated following Lu et al.^60^ which uses both electric fields and the wind velocity.

The solar wind and interplanetary magnetic field data are obtained from the WIND spacecraft located at L1 Lagrangian point via https://wind.nasa.gov/data.php. All data are averaged into 92 seconds resolution. The 1-minute modified SYM-H index is from OMNIweb (https://omniweb.gsfc.nasa.gov/). OMNIweb also provides 1-minute modified solar wind and interplanetary magnetic field data shifted to Earth’s bow shock nose involving ACE, Wind, IMP8 and Geotail satellites. It is to be noted that OMNI data introduces large errors in the data propagation scheme^61^.

## Results

**Figure 1:**
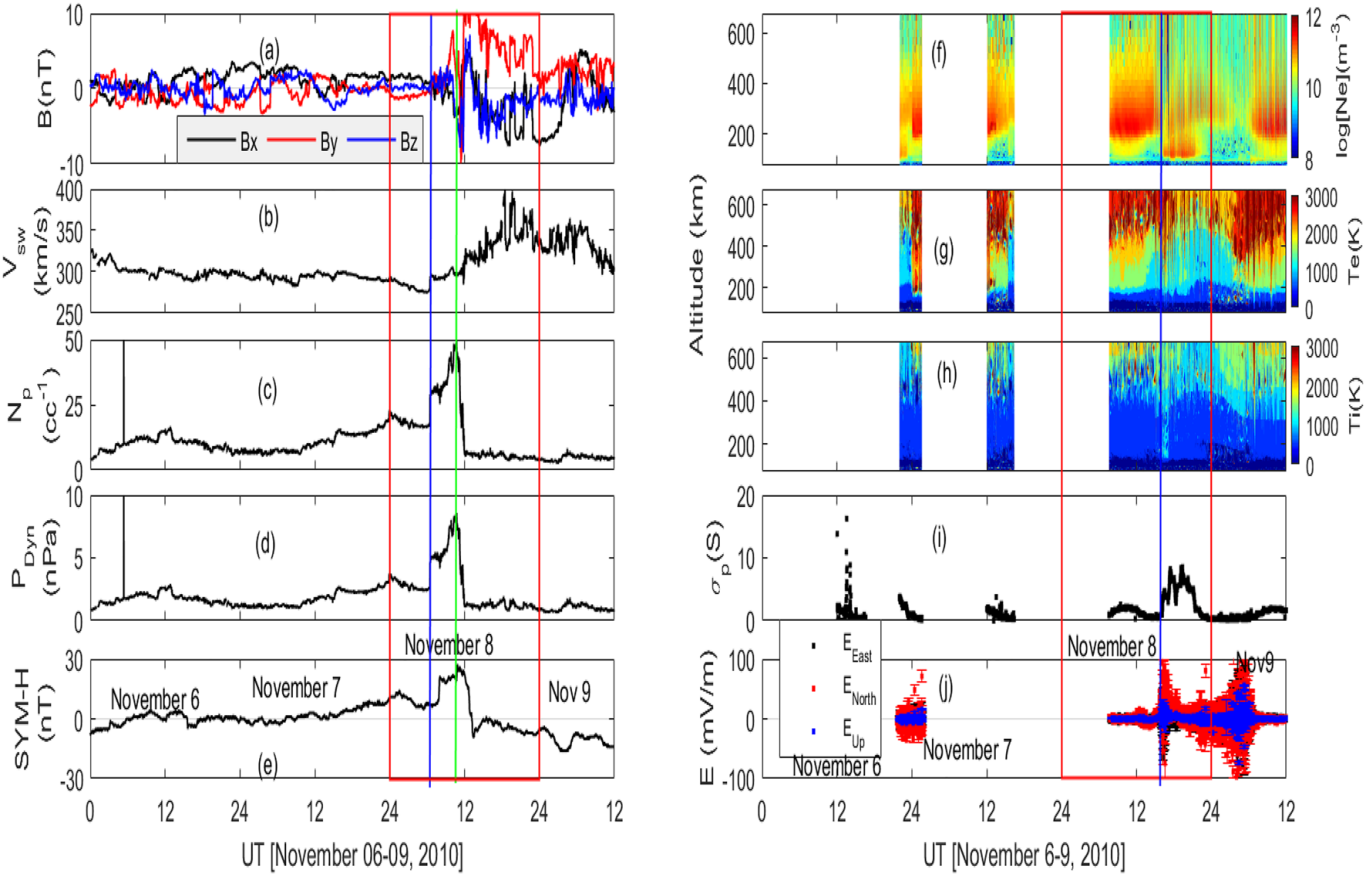
Time evolution of solar wind and interplanetary magnetic field (IMF) data and EISCAT measurement during November 06–09, 2010 (Red box shows variation during November 08, 2010). (**a**) IMF, (**b**) solar wind speed, (**c**) solar wind density, (**d**) solar wind dynamic pressure, (**e**) SYM-H index, EISCAT measurements of (**f**) electron density, (**g**) electron temperature, (**h**) ion temperature, (**i**) Pederson conductance and (**j**) electric fields. Solar wind and IMF data are from WIND spacecraft. SYM-H index is obtained from OMNIweb. The blue and red vertical lines on the left panels, respectively, represent the onset time of increase and decrease dynamics pressure. On the right panels, the vertical blue line represents the time of increase in the Pederson conductivity, electric field and low altitude electron/ion temperature.

The time variation of (a–e) solar wind and interplanetary magnetic data from the WIND spacecraft and (f–j) EISCAT measured electron density, temperature, Pederson conductance and the electric fields during November 06–09, 2010 are shown in Fig. 1. The SYM-H index is from OMNIweb. The solar wind and interplanetary magnetic field (IMF) showed a smooth variation prior to November 08, 2010 (Fig. 1a–e). The variation during November 08, 2010 is shown rectangular box. A significant variation is observed in the solar wind parameters at around 8 UT on November 08, 2010 (represented by a vertical blue line). The IMF display fluctuations with directional change. Whereas, a sharp increase is noticed in the solar wind speed, density and dynamic pressure. IMF By and Bz, respectively, reached the minimum values of $-10$ nT and $-8$ nT, at around 13 UT. The solar wind speed increased continuously till about 20 UT. The density and dynamic pressure stayed elevated for about four hours. The density (Np) dropped by about 90$\%$ from the peak value of 50.5 cc$^{-1}$ to 5.1 cc$^{-1}$ within 1.5 h which is represented by a green vertical line. Similar higher number density has also been reported earlier. For example, Belakhovsky and Vorobjev^62^ reported a change of Np from 35 to 10 cc$^{-1}$ during negative sudden impulse on September 28, 2009. During 17 March 2015 dynamic pressure pulse event, density reached the value of 58 cc$^{-1}$^63^. Study by Ni et al.^64^ observed a maximum density of 31.3 cc$^{-1}$ on June 7, 2014. It is to be noted that Fogg et al.^65^, by using a 5 years of solar wind density from OMNI, observed solar wind density as 4.46 cc$^{-1}$ with a standard deviation of 5.136 cc$^{-1}$. A sudden decrease can also be noticed in the dynamic pressure that dropped from 8.6 nPa to 1.0 nPa. The depletion in the solar wind density and pressure, and increase in the speed and IMF suggests the slow reverse shock as the driver of this event^1^. The SYM-H index also exhibits an identical behavior with minimum value of $-9$ nT at 1330 UT. In response to the sudden changes in the solar wind and IMF, significant variations can be observed in the EISCAT measured ionospheric parameters at Troms${\o }$, Norway, about 5 h after the sudden drop in pressure and density. The electron density shows a depletion (enhancement) in the higher (lower) altitude region that lasted from 16 to 22 UT on November 08, 2010 (Fig 1f). The onset time of this change is represented by a vertical blue line. The electron/ion temperature, Pederson conductance and electric fields demonstrate strong increments during the same period (Fig. 1g–j). The increase in the electron/ion temperature can particularly be noticed in the lower altitude (<300 km) region at around 16 UT (see vertical blue line). The Pederson conductance shows a sharp increase at around 16 UT with a peak value of about 8.5 Siemen about 3–4 h later. The electric fields display strong fluctuations (Fig. 1j).

**Figure 2:**
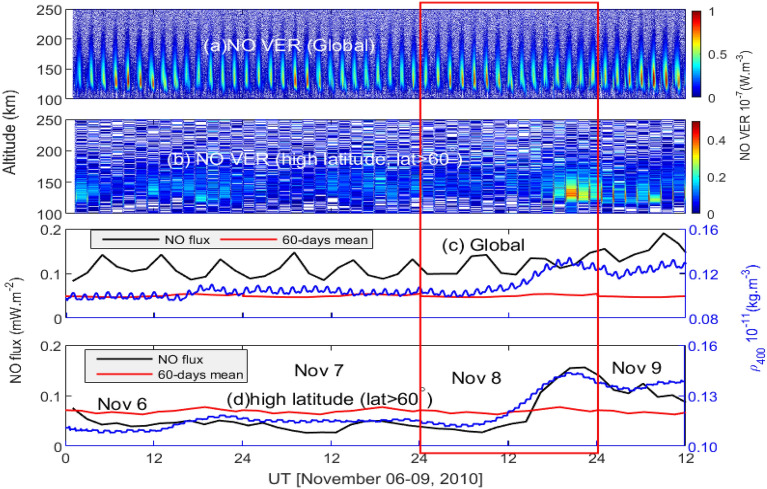
Altitude-time cross-section of NO volume emission rate, (**a**) global, (**b**) high latitude (latitude > $\>60^\circ$ N) variation. NO flux (black color) with 60-days mean (red color)and thermospheric density (blue color) normalized to 400 km, (**c**) global and (**d**) high latitude variations.

The global variations of altitude-time cross-section of the TIMED/SABER satellite observed NO volume emission rate (VER) is depicted in Fig. 2a. The global variation includes all available data during the concerned period. During November 06–09, 2010, TIMED/SABER was in the north-view mode and covered the 83$^\circ$ N to $-53^\circ$ S latitude. Consequently, only high latitude (latitude >60$^\circ$ N) region in north hemisphere is considered for high latitude variations. The high latitude variation shows an increment during 16–24 UT on November 08, 2010. The NO VER is integrated vertically to get cooling flux. The global variations of 2-h averaged NO flux, Gravity Recovery and Climate Experiment (GRACE)-A satellite^66^ measurement of thermospheric density along with 60-days averaged NO flux are shown in Fig. 2c. The GRACE-A satellite measurements of thermospheric density is normalized to 400 km using NRLMSISE-00 model^44^. A significant increase in the thermospheric density can be observed post negative pressure impulse period. It could be due to the fact that the GRACE-A satellite covers high latitude regions in both the hemispheres. The high latitude variation is shown in Fig. 2d. The high latitude thermospheric density increases by about 30$\%$ reaching the maximum value of 0.145$\times 10^{-11}$ kg m$^{-3}$ at around 18–19 UT. NO flux increases about five times from the pre-event value of 0.035 mW m$^{-2}$. The peak NO flux lags the thermospheric density by about 2–3 h.

**Figure 3:**
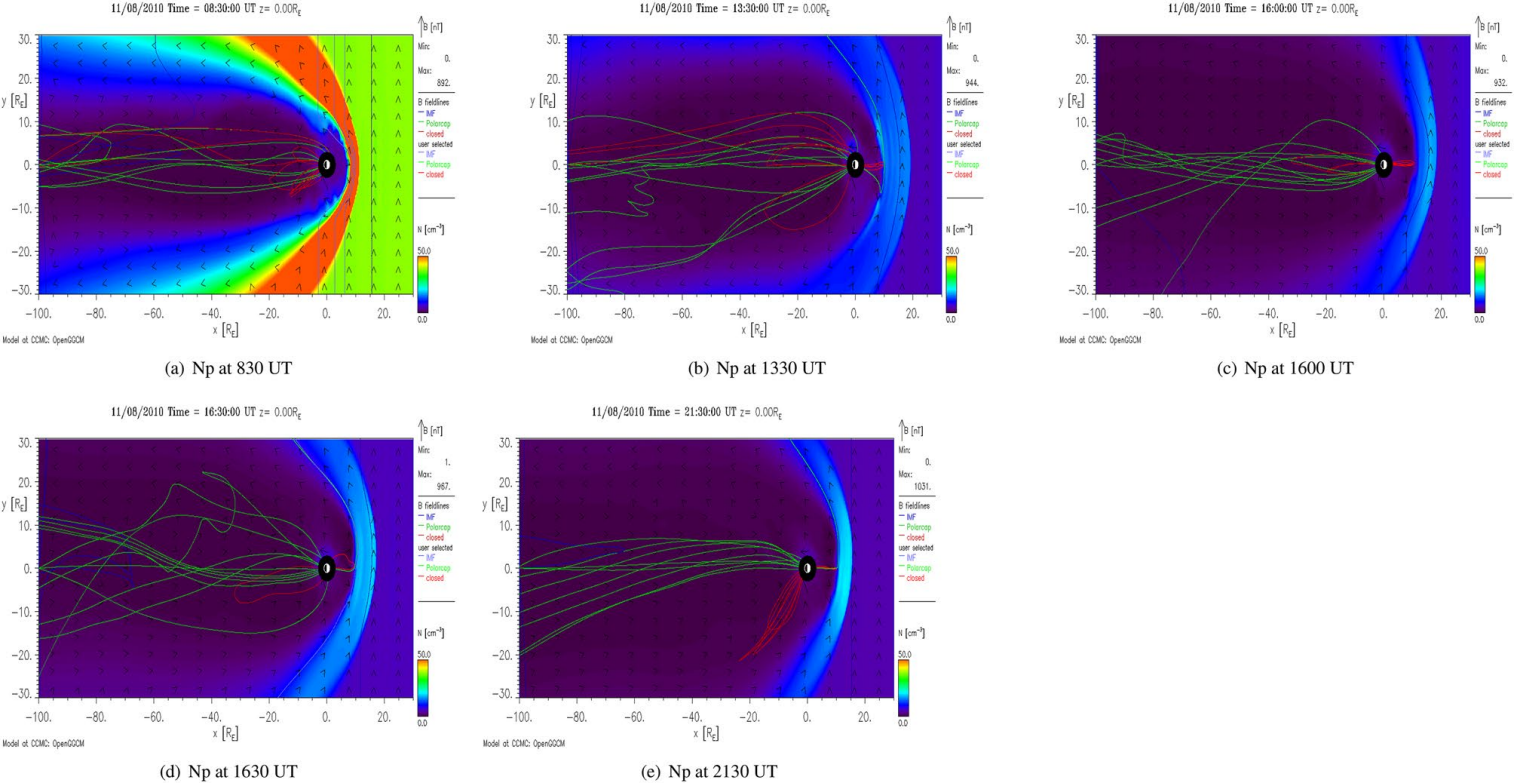
Cross-sectional view of OpenGGCM simulated number density (Np) at (**a**) 830 UT, (**b**) 1330 UT, (**c**) 1600 UT, (**d**) 1630 UT and (**e**) 2130 UT during November 8, 2010 as obtained from NASA’s Community Coordinated Modeling Center.

**Figure 4:**
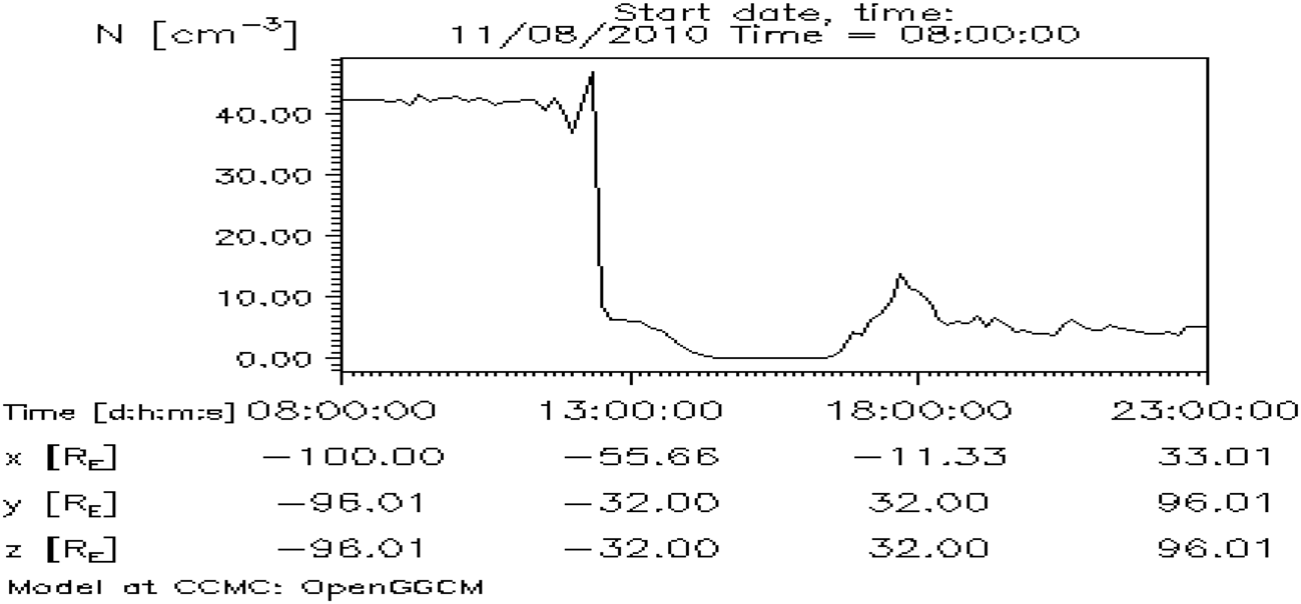
Time variation of number density (Np) during November 8, 2010 as obtained from NASA’s Community Coordinated Modeling Center.

Figure 3 shows the OpenGGCM simulated number density across the magnetosphere at five different times (0830, 1330, 1600, 1630 and 2130 UT) during November 08, 2010 in x–y plane as obtained from the visualization of 3D data at NASA’s Community Coordinated Modeling Center. The number density around magnetosphere at 830 UT density is depicted in Fig. 3a. The stand-off distance of magnetopause is located around 10R$_E$ on the sunward direction. The number density drops abruptly at around 1330 UT resulting in the expansion of the magnetosphere with compressed magnetic field lines. The number density decreases further at 1600 UT (Fig. 3c). Subsequently, the magnetosphere expanded further with entangled magnetic field lines. Figure 3e shows the compressed magnetosphere (as compared to 1600 UT) at 2130 UT due to increase in the density. The temporal variation of the number density (Np) is shown in Fig. 4.

**Figure 5:**
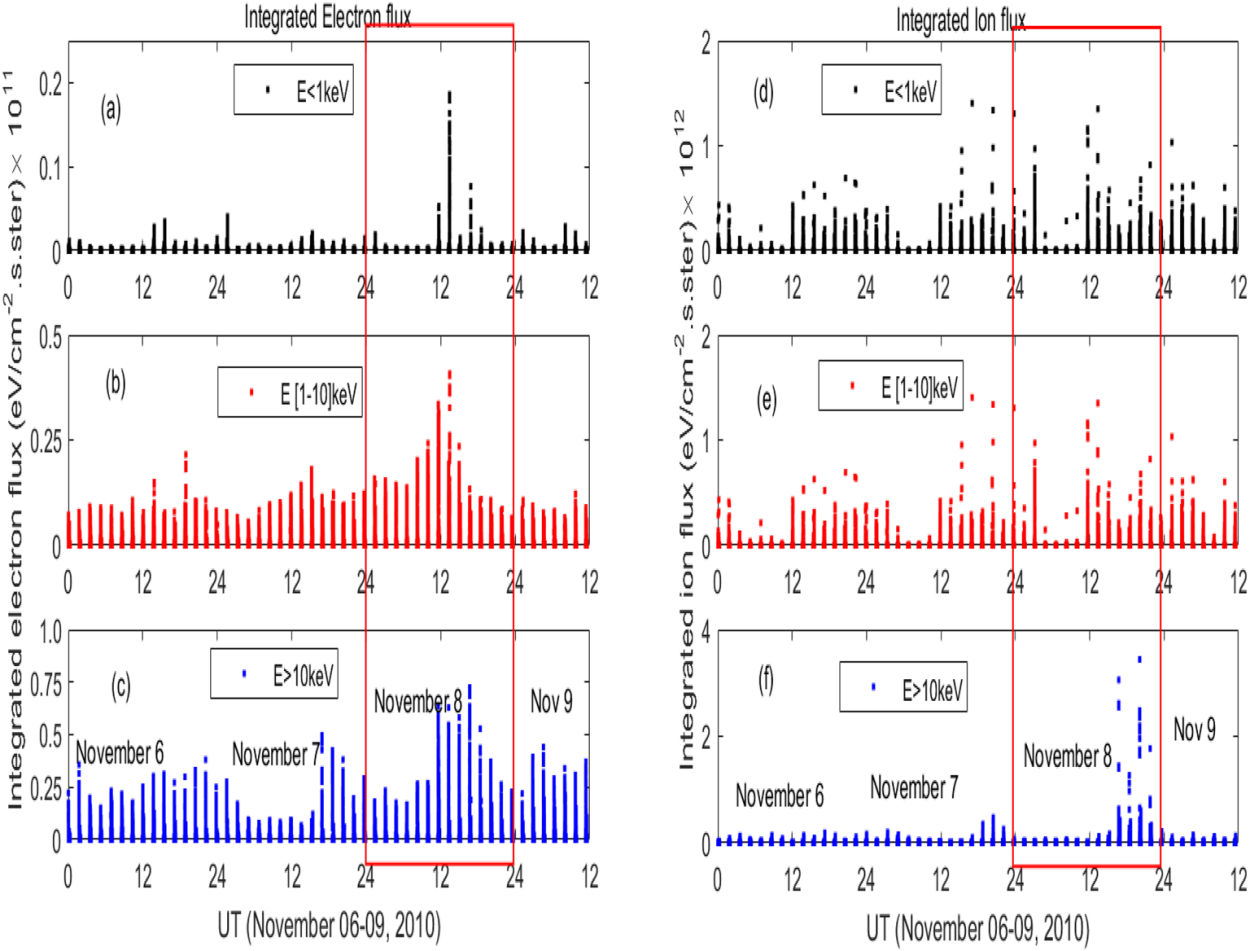
Time variation of integrated particle flux from DMSP satellite for different energy ranges [left panels (**a–c**): electron flux, right panels (**d-f**): ion flux] during November 06–09, 2010; variation during November 08, 2010 is boxed.

In order to understand the variation in the low energy particle flux, we present integrated particle flux during November 06–09, 2010 in Fig. 5. The particle flux data are obtained from the Defense Meteorological Satellite Program (DMSP) F18 satellite via the Madrigal database (http://cedar.openmadrigal.org). DMSP has low energy particle detector sensor to measure auroral precipitating particle from 30 eV to 30 keV in 20 energy channels^67^. The electron and ion flux are divided into three categories; low ($<1$ keV), mid (1–10 keV) and high (>10 keV). Corresponding variation during November 08, 2010 is inset. Both the electron and ion flux show pre-event enhancements beginning at around 12 UT on November 08, 2010. However, ion flux of higher energy (>10 keV) increases at later time (Fig. 5f).

**Figure 6:**
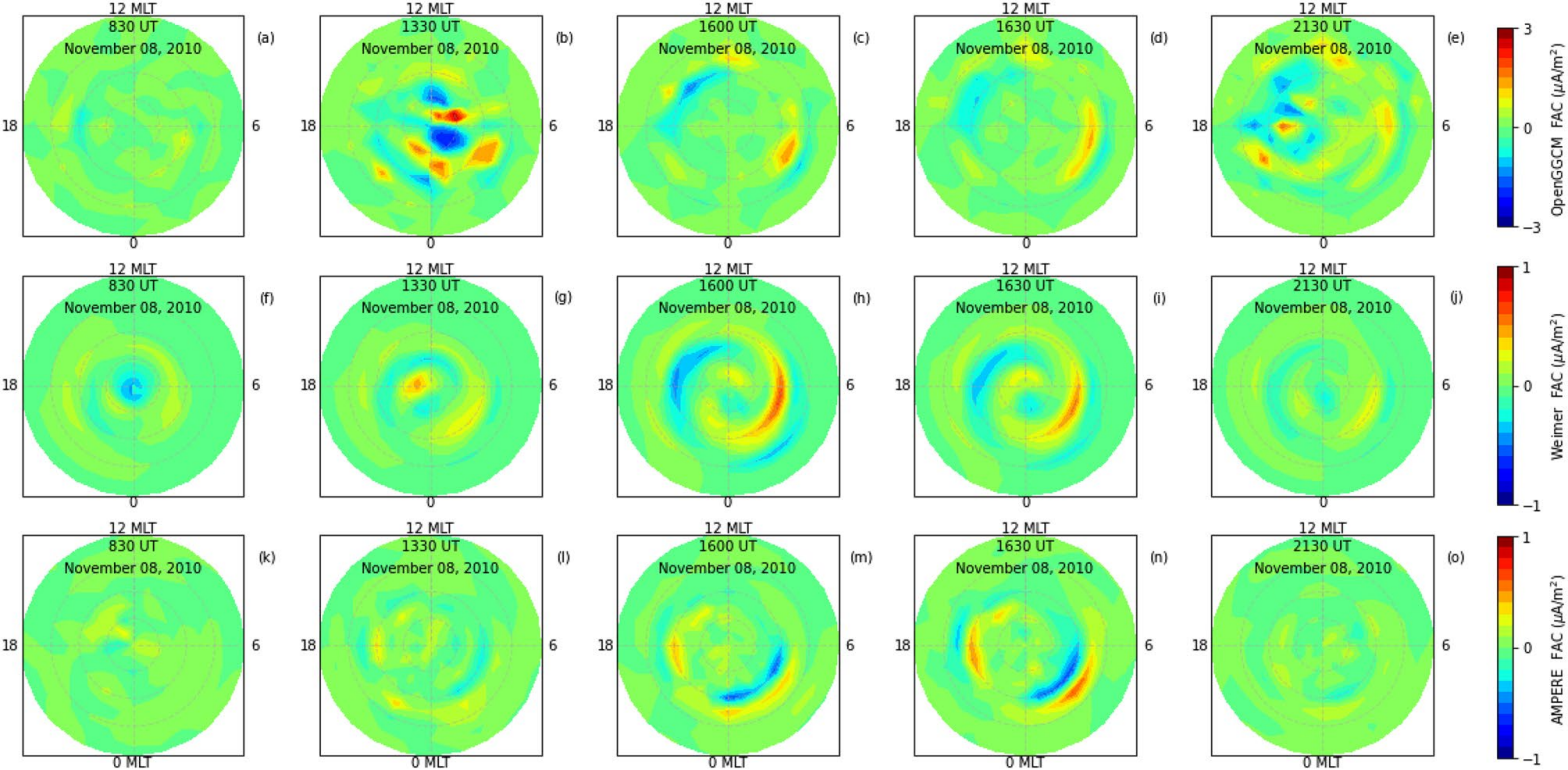
Field-aligned-currents from (**a–e**) OpenGGCM simulation, (**f–j**) Weimer model and (**k–o**) AMPERE observations at 830 UT, 1330 UT, 1600 UT, 1630 UT and 2130 UT on November 08, 2010 over northern hemisphere.

Figure 6 shows the field-aligned-currents (FACs) from the models and observations at five different times on November 08, 2010 over north hemisphere. The leftmost and rightmost panels are, respectively, the variations at 830 and 2130 UT. The top (a–e), middle (f–j) and the bottom (k–o) panels are, respectively, the FACs from the OpenGGCM simulations, Weimer model and AMPERE observations. The AMPERE observations are 10 minutes averaged FACs centered around mentioned periods. The OpenGGCM simulation results in significantly higher FACs as compared to the Weimer model and AMPERE observations. Weimer model displays a weak region 1 (R1) and region 2 (R2) FAC at 830 UT. No appreciable variation is noticed in the OpenGGCM simulation and AMPERE observation. The OpenGGCM and the AMPERE observations show the enhanced and discrete FACs localized around the evening-noon sector at 1330 UT. The Weimer simulated FACs are collocated on magnetic latitude-magnetic local time cross-section. Both modeled and observed FACs are intensified with equatorward expansion during post negative pressure impulse period. The strongest FACs from OpenGGCM and AMPERE observations are pronounced at 1630 UT. Whereas, the Weimer modeled FACs are strongest at 1600 UT. In addition, it can be noticed that AMPERE observations of R1 and R2 FACs have almost opposite parity as compared to the simulations.

**Figure 7:**
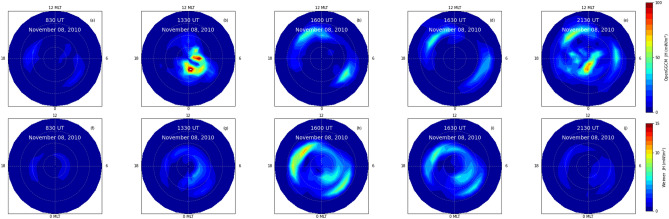
Joule heating rates from (**a–e**) OpenGGCM simulation and (**f–j**) Weimer model at 830 UT, 1330 UT, 1600 UT, 1630 and 2130 UT on November 08, 2010 over northern hemisphere. Note Joule heating rate from the OpenGGCM at 1330 UT is divided by 3.

The model simulated Joule heating rate (JH) over north hemisphere is shown in Fig. 7 at different times on November 08, 2010. The OpenGGCM simulated JH are depicted on the top panels, whereas the bottom panels correspond to the Weimer model. Weak JH is observed at 830 UT (leftmost panels). The OpenGGCM simulation shows an exceptional enhancement at 1330 UT; note JH is multiplied by 1/3 (Fig. 7b). The JH, from the both the OpenGGCM and Weimer models, demonstrates an increment and equatorward expansion in response to the negative pressure impulse. This enhancement is more pronounced in the evening/morning sector that reached to about 60$^\circ$ magnetic latitude. However, it can be noticed the OpenGGCM shows a discrete variation at 2130 UT unlike the Weimer JH (Fig. 7e). The Weimer model exhibits more a systematic variation with a maximum at 1600 UT. It has relatively more latitude and local-time coverage as compared to the OpenGGCM. Further, two band structures, located around 75$^\circ$ and 65$^\circ$ latitudes, in the midnight-dawn sector can be noticed in the Weimer modeled JH. The discrepancy between these two model results can be attributed to the fact that Weimer is an empirical model based on previous satellite measurements to specify the ionospheric field aligned current, whereas, OpenGGCM is a time dependent MHD model that considers both the magnetospheric and ionospheric aspect of field aligned currents. The detailed investigation is out of scope of the current study.

**Figure 8:**
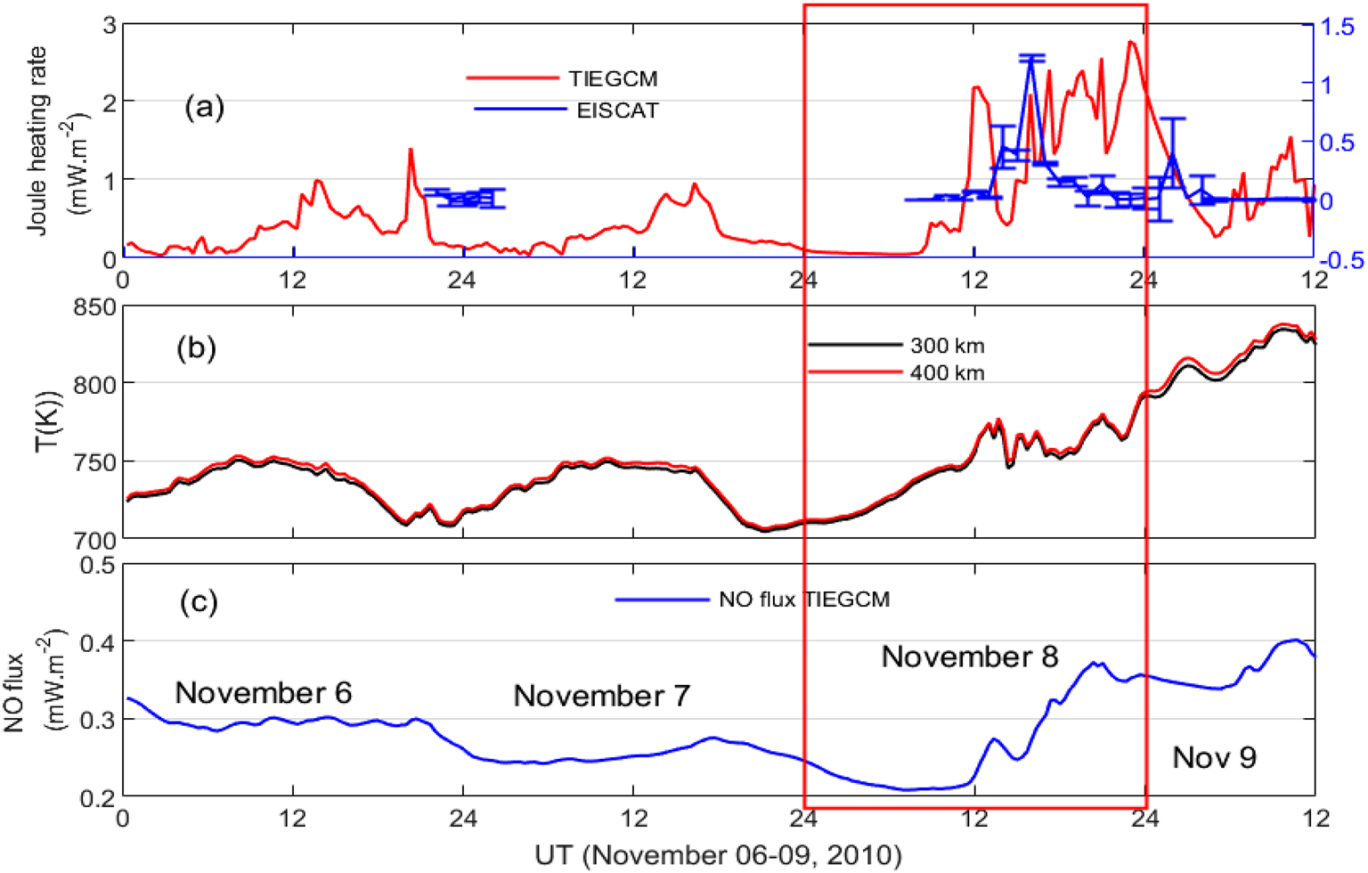
Time variation of Joule heating rates from (**a**) EISCAT measurement (blue color) and the TIEGCM simulations (red color), TIEGCM simulations of (**b**) temperature and (**c**) nitric oxide cooling flux during November 06–09, 2010 for latitude > $\>60^\circ$ in the northern hemisphere; November 08, 2010 is boxed.

Figure 8 shows the temporal variations of the EISCAT measured JH and the TIEGCM simulated JH along with temperature and NO cooling flux over high latitude in north hemisphere. Both the EISCAT measurements and the TIEGCM simulations show substantial enhancements during November 08, 2010. The EISCAT JH peaks at around 1600 UT with peak magnitude of about 14.9 mW m$^{-2}$. Whereas, the TIEGCM shows strong temporal fluctuations during 12–24 UT period. The TIEGCM temperature and the NO cooling flux also undergo significant increments with sharp increase at 12 UT on November 08, 2010 (Fig. 8b, c).

**Figure 9:**
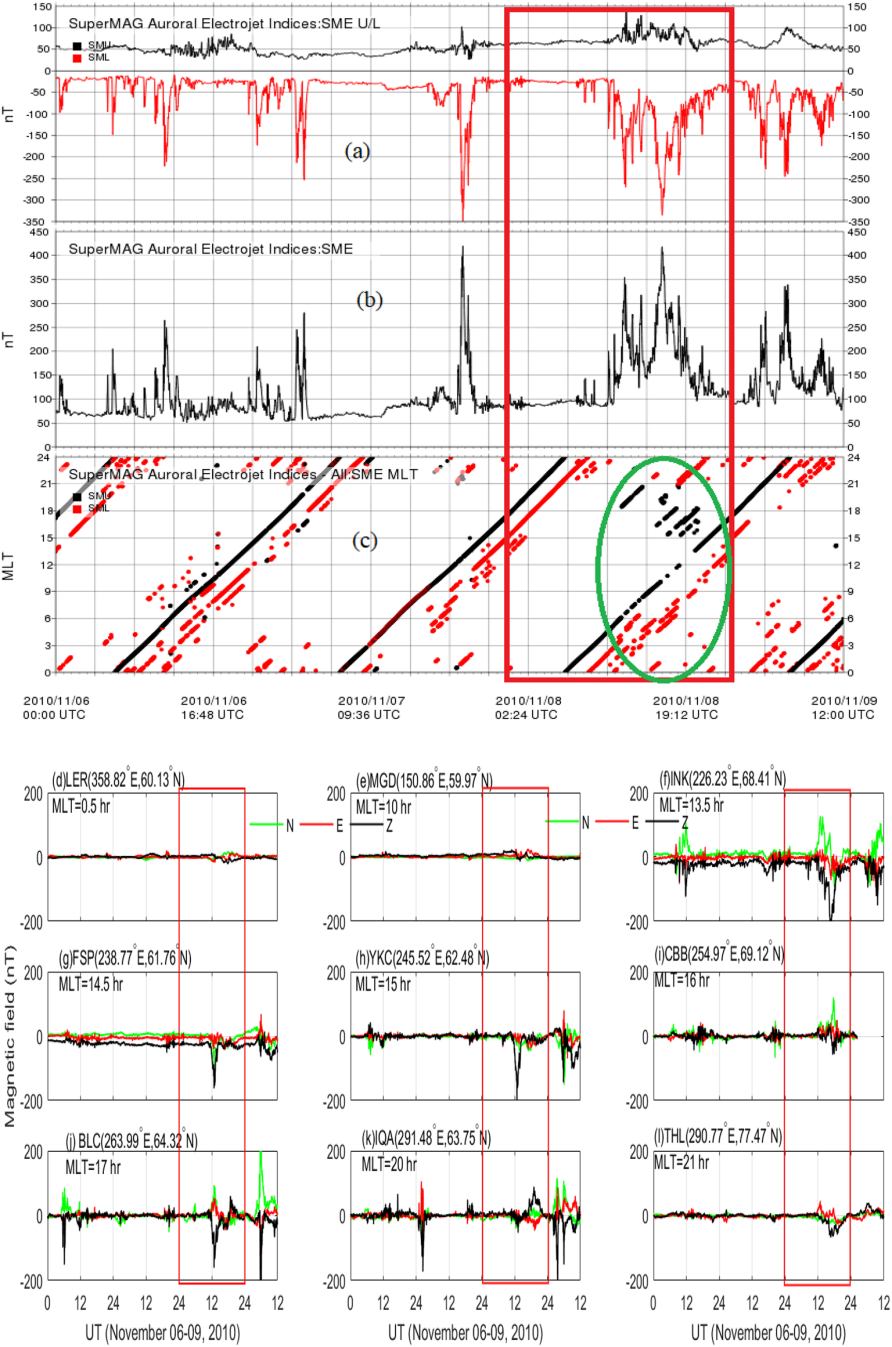
Temporal variation of AE index and magnetic fields. (**a**) AU/AL index, (**b**) AE index, (**c**) MLT-Time cross-section and (**d–l**) magnetic field at different MLT sectors, as obtained from SuperMAG. Variation during November 08, 2010 is boxed. SME, SML and SMU indices are, respectively, equivalent to AE, AL and AU indices.

In order to investigate the delayed response of Joule heating to the negative SI event, we use temporal variation of Auroral Electrojet(AE) along with the magnetic local time(MLT)-time cross-sectional view and the ground magnetometer data at different MLT sectors as obtained from SuperMAG database via https://supermag.jhuapl.edu and are presented in Fig. 9. AU($\simeq$SMU) and AL($\simeq$SML) indices are, respectively, denoted by black and red color (see Fig. 9a, c). Both AU and AL indices increase significantly post-SI period. AU index is already elevated as compared to previous day (Fig. 9a). However, it begins to increase at around 11 UT on November 8, 2010 and stays elevated till about 1930 UT. AU index reaches the peak magnitude of about 140 nT at around 1300 UT. Similar variation with relatively higher magnitude can also be noticed in AL index which peaks at 1700 UT with the magnitude of $-340$ nT. This increase in AU/AL index strengthens both eastward and westward currents resulting in the strong intensification of AE ($\simeq$SME=SMU-SML) index with peak value of 360 nT at around 1700 UT (Fig. 9b). The MLT-time cross-sectional view of AU/AL index is shown in Fig. 9c. An unexpected increase in the AU (black color) and AL (red color) indices can be observed, respectively, in the afternoon/evening and midnight/morning MLT sectors during November 8, 2010 (Fig. 9c, encircled; see the variations away from the diagonal lines) with a significant time delay between SI event and peak AE. The deviation from the main diagonal line suggests the MLT of the dominant activity. This behavior can also be inferred from the magnetometer data which shows early variations in the afternoon/evening sector (Fig. 9d–l).

## Discussions

We investigate the response of thermospheric NO 5.3 $\upmu$m emission to the negative pressure impulse during November 08, 2010. The sudden decrease in the solar wind density associated with IMF at around 1200 UT on November 08, 2010 resulted in the sharp decrease in the solar wind pressure from about 8.6 nPa to 1.0 nPa. The Kp index was stable below 2+ throughout November 06–09, 2010. A sudden decrease in the SYM-H index was observed due to the abrupt depletion of the dynamic pressure and the magnetopause current^27,68^. In response to the negative pressure impulse, the thermospheric cooling emission and density show strong enhancements particularly in the high latitude region (Fig. 2).

The thermospheric cooling has both direct and indirect production sources. The abundance of the nitric oxide, atomic oxygen and thermospheric temperature directly affect the variability of NO cooling emission. Whereas the low energy particle precipitation indirectly dictates the behavior of NO cooling emission by increasing the production of NO density. As it is well known that the NO emission accounts for the majority of Joule heating energy during geomagnetic active period, we also investigate both the aspects.

The negative and positive pressure impulses, respectively, expand and contract the magnetosphere by increasing the magnetic reconnection on dayside (Fig. 3). The sudden expansion of dayside magnetosphere is known to form discrete aurora due to low energy particle precipitation by pitch angle diffusion and Field-Aligned-Current^8,28, 29, 69^. Both the modeled and observed FACs display significant enhancements with equatorward expansion during negative pressure impulse period (Fig. 5). It also generates the ionospheric flow vortex and equatorward expansion of auroral boundary. The flow vortex and auroral arc strongly affect the electron density and electron/ion temperature^70,71^. In addition, the equatorward expansion of auroral oval due to the southward turning of IMF Bz also increases the electron/ion temperature which can be noticed in Fig. 1.

The temporal variation of the low energy particle precipitated into high latitude region is depicted in Fig. 5. The integrated electron flux shows a strong enhancement during negative pressure impulse. The precipitated particles impact the ionosphere–thermosphere system^72^. Earlier study reported that electrons of energy less than 1keV significantly affect the cooling emission and upliftment of thermospheric density^72,73^. The electron flux of energy range 1.4–30 keV is the dominant contributor of thermospheric cooling emission with strongest in the energy range of 1.4–3.1 keV^73^. The electrons of 1keV energy creates NO density in the altitude above 120 km. The electrons of energy 1.4–4.6 keV can deposit energy in the altitude region of 100–110 km^34,35^. The ions also strongly contributes to the production of NO density. The study by Galand et al.^74^ shows that the ion flux of 1–20 keV can produce more than 50$\%$ of NO density in the night atmosphere. The cooling by proton accounts for 30$\%$ of total cooling due to electrons, and about 1/4th of total cooling^73^. It is due to the dissociation of N$_2$ molecules in to N($^4$S), and N($^2$D) by auroral electrons and ions. These nitrogen atoms, further, in reaction with molecular oxygen density form NO molecule^33–35^. The strong increase in the particle flux would result in the higher production NO density and NO cooling flux (Fig. 2). It is evident the fact that a slow reverse shock impacted on magnetosphere can be significantly geoeffective. The variation of high energy particle flux during fast reverse shock, by using multi-satellite observations and numerical simulations, has been studied by Bhaskar et al.^75^. Their study shows a decrease in the magnetic field and energetic particle flux ($\sim$ 40–475 keV) as observed by spacecrafts residing on dayside that propagates towards the nightside magnetosphere. Further, they conclude that particle exhibits a non-dispersive response to shock.

By using ACE solar wind data, AE index and GUMICS MHD simulation, Palmroth et al.^76^ reported an enhancement in the Joule heating rate in associated with the positive pressure impulse during southward IMF. They demonstrated that R1 FAC controls the Joule heating rate during increased dynamic pressure via Chapman-Ferraro current system. Similarly, the R2 FAC affects the Joule heating rate via inner magnetospheric pressure. It is because there is a pressure balance between the lobe field and solar wind dynamic pressure, and lobe field and the plasma sheet. The dynamic pressure would result in the inner magnetospheric pressure increment and decrease in the plasma sheet. Fogg et al.^21^, by using multi-instrument observations during positive pressure impulse event on June 16 2012, concluded that the effect of interplanetary magnetic field is dominated by Bz component. It is to be noted that the Joule heating rate during negative pressure impulse period is yet to be explored to the best of our knowledge. The OpenGGCM and the Weimer model simulations show strong enhancement of Joule heating rate during negative pressure impulse period (Fig. 7). It can be attributed to the increase FACs which strong positively correlated with the Joule heating rate^76^. Similar strong enhancement in the Joule heating rates can also be observed from the EISCAT measurement at Troms${\o }$, Norway, and the TIEGCM simulation (Fig. 8a). The TIEGCM simulations are shown for northern hemispheric high latitude region. It is well known that the Joule heating rate can increase the temperature, composition and density (Fig. 8b). In order to regulate the thermospheric temperature and Joule heating rate, the thermospheric cooling shows a strong enhancement because of its thermostat nature which converts kinetic energy to infrared energy that exits the thermosphere which is clearly noticed in Fig. 8c. The understanding of thermospheric NO cooling enhancements during geomagnetically active times is important for thermospheric neutral mass density models because it can be a significant source of errors in these models^77^, even ones caused by a slow reverse shock. We would like to mention here that earlier studies report an immediate response of the magnetosphere–ionosphere system to negative SI. However, in the present study, we observed an unexpected high delayed response of MIT system. It can be attributed to the fact that the current system takes time to build-up which dictates the Joule heating rate(=2.3 $\times$ 10$^8$ AE^79^). The eastward current (represented by AU index) is strongest in the afternoon/evening sector. The westward current (represented by AL index) is strongest in the midnight/morning sector (see Fig. 9a–c). It results in the peak AE index at around 1700 UT. Subsequently, a delay is expected in the Joule heating rate. In addition, we would like to emphasize here that the delayed response of MIT might add to doubt the event as “SI”. Nevertheless, there exists significant contention regarding its characterization, and the definition remains the subject of an ongoing debate.

## Conclusions

We, for the first time, investigate the response of NO emission to negative pressure impulse driven by slow reverse shock during a geomagnetically quiet period of November 06-09, 2010. The TIMED/SABER satellite measurements of NO cooling emission along with the numerical model simulations and observations of Field-Aligned-Current, Joule heating rate and low energy particle flux are utilized. The negative pressure impulse drives an intensification and equatorward movement of Field-Aligned-Current which prompts low energy particle precipitation and Joule heating. Both particle precipitation (indirectly) and Joule heating (directly) dictate the variations in NO cooling emission. The amplification of precipitated particle flux increases the production of NO emission due to the formation of higher NO density. NO emission increases in order to regulate large energy perturbations due to Joule heating. The present study shows the evidence that a slow reverse shock impacted on magnetosphere can be significantly geoeffective. Further, the enhanced response of NO cooling emission to the negative pressure impulse during this geomagnetic quiet event underscores the role of thermospheric cooling in influencing space conditions. Moreover, the recent loss of Starlink satellites in February 2022^78^ highlights the ongoing relevance of understanding and monitoring these atmospheric variations for satellite operations and space mission planning.

## Data Availability

The SABER data were downloaded from SABER website via (https://saber.gats-inc.com/data.php). We acknowledge the Community Coordinated Modeling Center (CCMC, https://ccmc.gsfc.nasa.gov/) at Goddard Space Flight Center for the use of the OpenGGCM, Weimer and TIEGCM; Run numbers: tikemani_bag_101223_2(OpenGGCM), tikemani_Bag_101223_IT_1 (Weimer), and TIkemani_Bag_111923_IT_1 (TIEGCM). The AMPERE Field-Aligned-Currents are obtained via https://ampere.jhuapl.edu/. DMSP particle flux are from Madrigal database via http://cedar.openmadrigal.org. EISCAT data are obtained via https://eiscat.se/. The AE and magnetometer data are from SuperMAG via https://supermag.jhuapl.edu. The solar wind parameters are obtained from the WIND spacecraft via https://wind.nasa.gov/data.php. SYM-H index is from OMNIWeb via (https://omniweb.gsfc.nasa.gov).
